# Predictors of Short-Term Outcomes in Living Donor Renal Allograft Recipients: A Prospective Study From a Tertiary Care Center in North India

**DOI:** 10.7759/cureus.28335

**Published:** 2022-08-24

**Authors:** Elenjickal Elias John, Sudhir Mehta, Preet Mohinder Sohal, Jasvinder Singh Sandhu

**Affiliations:** 1 Nephrology, Christian Medical College, Vellore, Vellore, IND; 2 Nephrology, Maharishi Markandeshwar Institute of Medical Sciences and Research, Mullana, IND; 3 Nephrology, Dayanand Medical College and Hospital, Ludhiana, IND

**Keywords:** graft rejection, induction therapy, estimated glomerular filtration rate (egfr), post-transplant diabetes mellitus, spousal transplant, short-term transplant outcomes, renal allograft recipient, related donors, graft dysfunction

## Abstract

Background

Renal transplantation is the optimal treatment for patients of all ages with end-stage kidney disease. The long-term outcomes of renal transplantation are assessed by graft and patient survival rates. These outcomes are, in turn, influenced by post-transplant events such as delayed graft function, rejections, post-transplant infections, and post-transplant diabetes mellitus (PTDM). Each of these short-term outcomes is, in turn, determined by the interplay of various factors in the pre-, peri-, and post-transplant period. This prospective study was designed to understand the factors affecting short-term outcomes in living donor transplantation and their effect on graft and patient survival.

Methodology

A total of 86 patients underwent live donor renal transplantation between January 1, 2015, and March 31, 2016, at a tertiary care hospital in north India. Of these, five were lost to follow-up, and the remaining 81 patients were prospectively followed up to December 31, 2017.

Results

The majority of the recipients were males (91%) and the donors were females (74%). Spousal and related donors comprised 49% and 51% of donations, respectively. The mean estimated glomerular filtration rate (eGFR) of donors was 98 ± 9.2 mL/minute/1.73m². Induction therapy with basiliximab was given to 21/81 (26%) recipients. The majority of recipients (68/81, 84%) received triple-drug immunosuppression with prednisolone, tacrolimus, and mycophenolate mofetil. Delayed graft function (DGF) occurred in 4/81 (4.9%) cases. Biopsy-proven acute rejections (BPARs) occurred in 15/81 (18.5%) cases, two-thirds of which were acute antibody-mediated rejections (ABMRs). During the follow-up period, 50 episodes of infections occurred in 35/81 (43.2%) recipients, with the most common being urinary tract infection (23/81, 28.5%). PTDM was diagnosed in 22/81 (27.2%) patients beyond six weeks of transplant. On multivariate logistic regression analysis, the most significant predictor of DGF was acute rejections and vice versa. Acute rejections also predicted the occurrence of post-transplant infections. Pre-transplant hepatitis C virus (HCV) infection and cyclosporine-based therapy were significant predictors of PTDM. At the six-month follow-up, 10/81 (12.3%) patients developed graft dysfunction. The predictors of graft dysfunction at six months were recipients of related donors and rural patients. One-year graft survival, death-censored graft survival, and patient survival rates were 85.2%, 92.6%, and 91.3%, respectively. The most common cause of death was post-transplant infections (5/7, 71.4%) of which the majority (4/5, 80%) were fungal infections. On multivariate logistic regression analysis, the most significant predictor of graft loss and patient loss was low pre-transplant donor eGFR and PTDM, respectively.

Conclusions

Graft and patient survival in living donor kidney transplantation are influenced by a multitude of interdependent factors during the pre-transplant (donor eGFR, type of donor, socioeconomic status, HCV infection in recipient, type of immunosuppression) and the post-transplant (DGF, rejections, infections, and PTDM) period.

## Introduction

Short-term outcomes of renal transplants have improved because of a better understanding of transplant immunology, more effective and safer immunosuppressants, and improvisations of surgical techniques. Most of the data on the risk factors determining transplant outcomes were based on terminal events such as graft loss and patient loss which were mainly derived from retrospective studies done before 2010 [1-5]. These hard outcomes are, in turn, influenced by post-transplant events such as delayed graft function, rejections, post-transplant infections, and post-transplant diabetes mellitus (PTDM). Each of these short-term outcomes is, in turn, determined by the interplay of various factors in the pre-, peri-, and post-transplant period. With this background, this prospective study was conducted among our live-related renal allograft recipients to determine the effect of pre-transplant recipient and donor factors, post-transplant immunosuppression regimens, and infectious and non-infectious complications on the short-term outcomes.

## Materials and methods

Study design and population

This prospective observational study was conducted in the Department of Nephrology at a tertiary care hospital in north India between January 1, 2015, and December 31, 2017. All patients undergoing live donor renal transplantation between January 1, 2015, and March 31, 2016, were included. The study was cleared by the Institutional Ethics Committee on Human Research and approved by the Dayanand Medical College and Hospital (DM_216/589). The study complied with the Declaration of Helsinki of 1975, as revised in 2000. Informed written consent was obtained from all patients.

Baseline assessments and treatment

Detailed history and investigations into the etiology of renal disease, age, sex, duration of dialysis, body mass index (BMI), relationship with the donor, induction therapy, hepatitis B surface antigen (HbsAg), anti-hepatitis C virus (anti-HCV) antibody, anti-human immunodeficiency virus (anti-HIV) antibody, type of immunosuppressive therapy given, and urological, vascular, and infective complications were noted. High immunological risk renal transplants (those with complement-dependent cytotoxicity (CDC), flow cytometry crossmatch positivity, or recipients positive for donor-specific antibodies (DSA)) are not routinely done at our center and were excluded from this study. Induction therapy was offered to all recipients (low risk); however, due to financial constraints, only one-quarter of recipients received non-depleting interleukin 2 receptor blocker (IL2B), intravenous basiliximab 20 mg on the day of the transplant and on day four post-transplant. All patients were initiated on triple-drug immunosuppression (prednisolone, tacrolimus, and mycophenolate mofetil) in therapeutic doses which were adjusted based on trough levels. Mycophenolate was replaced by azathioprine in 9/81 (11%) recipients due to gastrointestinal intolerance (5/9) and persistent leucopenia (2/9). Tacrolimus was changed to cyclosporine in 6/81 (7.4%) recipients due to inadequate trough levels (5/6) and neurotoxicity (1/6). All recipients received three months of oral cotrimoxazole and valganciclovir prophylaxis.

Follow-up procedures and study outcomes

Regular check-ups of renal allograft recipients were done weekly in the first month and monthly for one year, and any complications noted were recorded and treated. All cases of biopsy-proven acute rejections (BPARs) were classified as per the 2019 modified Banff classification [6]. Protocol biopsies were not done. Various definitions used included “related transplants” - renal donors being either parents, siblings, children, grandparents, or grandchildren of the recipient; “spousal transplants” - renal donor either wife or husband of the the recipient; “dialysis vintage” - time between the initiation of dialysis and the date of transplant; “normal graft function” - adequate urine output and rapidly declining plasma creatinine levels after transplantation; “slow graft function” - plasma creatinine more than 3 mg/dL with no requirement of dialysis within one week of transplant; “delayed graft function” - dialysis needed in the first week of transplant; “acute allograft dysfunction” - rise in serum creatinine by more than 15% above the baseline level; “graft survival” - time between renal transplantation to either return to dialysis or transplant nephrectomy or repeat renal transplantation (whichever was earlier) censoring for death with functioning graft; “patient survival” - time between transplantation to death; “post-transplant diabetes mellitus” (PTDM) - new onset of diabetes mellitus after six weeks of transplant; “post-transplant erythrocytosis” (PTE) - plasma hemocrit more than 51% or hemoglobin more than 17 g/dL following kidney transplantation which persists for more than six months in absence of thrombocytosis, leucocytosis, or known cause for erythrocytosis. Response to anti-rejection therapy (ART) was classified as complete response (CR), serum creatinine level returns back to baseline level or is less than 1.2 mg/dL; partial response (PR) denoted the decline in serum creatinine level by more than 50% but does not return to baseline level or is more than 1.2 mg/dL; no response (NR) denoted worsening of renal function or decline in serum creatinine level by less than 50%.

Statistical analysis

Statistical analysis was done by SPSS version 25 (IBM Corp., Armonk, NY, USA). Categorical data were presented as percentages and quantitative data as mean ± standard deviation (SD) or median (range). Comparison of different groups was carried out using the chi-square test or Fisher exact test for categorical variables and independent Student’s t-test for comparing means of continuous variables. Multivariate logistic regression analysis was used to identify the predictors of various short-term outcomes. Survival analysis for time to graft loss and time to patient loss was done using the Kaplan-Meier test using the log-rank test for comparison between groups. Statistical significance was assumed at p-values of <0.05.

## Results

Baseline donor and recipient characteristics are listed in Table 1. A total of 86 patients underwent live donor renal transplantation between January 1, 2015, and March 31, 2016. Of these, five were lost to follow-up, and the remaining 81 patients were prospectively followed up to December 31, 2017. The most common cause of chronic kidney disease was chronic glomerulonephritis (43%). The majority of recipients (95.1%) were on maintenance hemodialysis for four (2-8.5) months prior to transplant. The majority of donations (70.4%) were from female donors to male recipients. Spousal donors comprised 40.9% of the donations, of which the majority (93.9%) were wives donating to their husbands. Among blood-related donors (59%), almost half of the donations were by mothers contributing to higher mean donor age compared to recipient age. Three-fourths of the donors had a pre-transplant eGFR of more than 90 mL/minute/1.73m². All donor recipient pairs were cross-match negative.

**Table 1:** Baseline characteristics of renal allograft recipients and renal donors. anti-HCV: antibodies to hepatitis C virus; BMI: body mass index; e-GFR: estimated glomerular filtration rate; N: number of cases; SD: standard deviation

Variable	Renal allograft recipients	Renal donors
Age (mean ± SD, years)	38.4 ± 13.4	48.3 ± 09
Sex (males/females, n (%))	74 (91)/07 (8.6)	21 (25.9)/60 (74.1)
BMI (mean ± SD, kg/m²)	23.4 ± 3.6	
Pre-transplant anti-HCV n (%)	8 (9.9)	0
Dialysis vintage (median (range), months)	4 (02–8.5)	
Prior blood transfusion, n (%)	27 (33.3)	
Prior renal transplant, n (%)	2 (2.5)	
eGFR (mean ± SD (range), mL/minute/1.73m²)		98 ± 9.2 (83–107)
Socioeconomic status n (%)		
Rural population	35 (43.2)	
Urban population	46 (56.8)	
Type of donor n (%)		
Related		48 (59.1)
Spousal		33 (40.9)

Infectious and non-Infectious complications post-transplant are presented in Table 2. DGF requiring dialysis in the immediate post-transplant period occurred in 4/81 (4.9%) cases. BPARs occurred in 15/81 (18.5%) cases, two-thirds of which were acute antibody-mediated rejections (ABMRs). Three-quarters of rejection episodes occurred in the first month of transplant. CR, PR, and NR to ART were observed in 9/15 (60%), 4/15 (26.6%), and 2/15 (13.3%) cases, respectively. During the follow-up period, 50 episodes of infection occurred in 35/81 (43.2%) recipients, with the most common being urinary tract infection (23/81, 28.5%). The most common causative agent was *Escherichia coli *(60.9%), followed by *Klebsiella *(21.7%) and *Enterobacter *(17.4%). PTDM was diagnosed in 22/81 (27.2%) patients beyond six weeks of the transplant. Of these, 5/22 (22.7%) had chronic HCV infection in the pre-transplant period.

**Table 2:** Infectious and non-infectious complications post-transplant. ABMR: antibody-mediated rejection; AZA: azathioprine; CMV: cytomegalovirus; CSA: cyclosporine; CVA: cerebrovascular accident; MMF: mycophenolate mofetil; N: number of cases; Pred.: prednisolone; PTE: post-transplant erythrocytosis; PTLD: post-transplant lymphoproliferative disease; PTDM: post-transplant diabetes mellitus; TAC: tacrolimus; TCMR: T-cell-mediated rejection; TMA: thrombotic microangiopathy; UTI: urinary tract infection

Variable	Total number of patients (%)
Induction therapy	
No induction	60 (74)
Basiliximax	21 (26)
Maintenance immunosuppression	
Pred. Tac MMF	68 (84)
Pred. Tac Aza	7 (8.6)
Pred. CSA MMF	4 (4.9)
Pred. CSA Aza	2 (2.5)
Urological complications	6 (7.4)
Graft pyonephrosis	2 (2.5)
Renal artery thrombosis/Lymphocele/Urinoma/Peri-renal hematoma	1 (1.2) each
Early graft function	
Normal graft function	65 (80.2)
Slow graft function	12 (14.8)
Delayed graft function	4 (4.9)
Rejections	15 (18.5)
Acute ABMR	10 (12.3)
Acute TCMR	2 (2.5)
Acute ABMR + TCMR	2 (2.5)
Chronic active ABMR	1 (1.2)
Post-transplant infections	
UTI	23 (28.5)
Tuberculosis	4 (5)
Other bacterial infections	7 (8.6)
Fungal infections	4 (4.9)
CMV	10 (12.3)
BK virus	2 (2.5)
Non-infectious complications	
PTDM	22 (27.2)
PTE	11 (13.5)
PTLD	1 (1.2)
Recurrence of native kidney disease	2 (2.4)
Causes of graft loss	
Rejection/Recurrence	2 (2.5) each
Renal artery thrombosis/Graft pyonephrosis/TMA	1 (1.2) each
Causes of death	
Infection	5 (6.2)
PTLD/CVA	1 (1.2) each

Significant predictors of short-term outcomes are listed in Table 3. On multivariate logistic regression analysis, the most significant predictor of DGF was acute rejections and vice versa. Acute rejections also predicted occurrence of post-transplant infections. Pre-transplant HCV infection and cyclosporine-based therapy were significant predictors of PTDM. At the six-month follow-up, 10/81 (12.3%) developed graft dysfunction. The predictors of graft dysfunction at six months were recipients of related donors and rural patients. One-year graft survival, death-censored graft survival, and patient survival were 85.2%, 92.6%, and 91.3%, respectively. The most common cause of death was post-transplant infections (5/7, 71.4%), of which the majority (4/5, 80%) were fungal infections. On multivariate logistic regression analysis, the most significant predictor of graft loss and patient loss was low pre-transplant donor eGFR and PTDM, respectively.

**Table 3:** Significant predictors of short-term outcomes in live related renal allograft recipients. anti-HCV: antibodies to hepatitis C virus; BPAR: biopsy-proven acute rejection; CI: confidence interval; CMV: cytomegalovirus; CSA: cyclosporine; DGF/SGF: delayed/slow graft function; eGFR: estimated glomerular filtration rate (mL/minute/1.73m²); PTDM: post-transplant diabetes mellitus; UTI: urinary tract infection

Predictors	Univariate P-value	Multivariable regression			
		Exp (B)	95% CI (upper)	95% CI (lower)	P-value
Predictors of DGF/SGF					
Donor age	0.010				
Induction therapy, No	0.008				
BPAR, Yes	<0.001	39.04	7.15	213.18	0.001
UTI, Yes	0.002	4.38	0.83	23.01	0.081
Urological complications, Yes	0.050				
Predictors of rejection					
Donor age	0.010				
Rural population, Yes	0.042	12.76	0.92	176.47	0.057
DGF/SGF, Yes	<0.001	39.57	6.15	254.63	0.001
Day 15 creatinine >1.5, Yes	<0.001				
First-month creatinine >1.5, Yes	<0.001				
Third-month creatinine >1.5, Yes	0.017				
PTDM, Yes	0.063				
Fungal infections, Yes	0.019				
UTI, Yes	0.005				
Predictors of graft dysfunction at six months					
Donor age	0.001				
Related donors, Yes	0.007	19.57	1.57	243.58	0.021
Rural population, Yes	0.022	5.32	1.03	27.54	0.046
No induction therapy, Yes	0.047				
DGF/SGF, Yes	0.008				
BPAR, Yes	0.028				
Bacterial infections, Yes	0.075				
First-month creatinine > 1.5, Yes	<0.001				
Third-month creatinine >1.5, Yes	<0.001				
Predictors of graft dysfunction at one year					
Related donors, Yes	0.050				
BK virus infection, Yes	0.049				
Third-month creatinine >1.5, Yes	<0.001	29.98	3.76	238.92	0.001
Six-month creatinine >1.5, Yes	<0.001				
Predictors of graft loss					
Donor age	0.003				
Recipient age	0.015				
Related donors, Yes	0.021				
Donor eGFR	0.018	0.90	0.81	0.99	0.046
CSA use, Yes	0.082				
Urological complications, Yes	0.057				
Third-month creatinine >1.5, Yes	0.014				
Six-month creatinine >1.5, Yes	0.001				
First-year creatinine >1.5, Yes	0.018				
Predictors of patient loss					
Female recipient, Yes	0.050				
Dialysis vintage	0.002				
BPAR, Yes	0.020				
PTDM, Yes	0.001	20.83	1.6	271.33	0.020
Bacterial infections, Yes	0.012				
CMV disease, Yes	0.037				
Fungal infections, Yes	<0.001				
Six-month creatinine >1.5, Yes	0.059	9.51	0.93	97.55	0.058
Predictors of post-transplant infections					
BPAR, Yes	0.001	4.64	0.99	21.76	0.052
No induction therapy, Yes	0.037				
DGF/SGF, Yes	0.001				
First-month creatinine >1.5, Yes	0.018				
Third-month creatinine >1.5, Yes	0.039				
Predictors of post-transplant diabetes mellitus					
Anti-HCV positive	0.031	6.57	1.30	33.31	0.023
CSA use, Yes	0.044	8.60	1.32	55.88	0.024
BPAR, Yes	0.063				
DGF/SGF, Yes	0.027				
Fungal infections, Yes	0.004				

Spousal versus related donors are presented in Table 4. Spousal donors were younger than related donors, and the majority were wives donating to husbands. There was no difference between the two groups in induction and maintenance immunosuppression protocols, early graft function, BPARs, and post-transplant infection rates. However, recipients of related transplants were at a higher risk of graft dysfunction at six months and one year, as well as graft loss.

**Table 4:** Spousal versus related donors. BPAR: biopsy-proven acute rejections; PTDM: post-transplant diabetes mellitus; SD: standard deviation

	Related donors (N = 48)	Spousal donors (N = 33)	P-value
Donor age (mean ± SD, years)	50.8 ± 8.9	44.7 ± 8.0	0.002
Recipient age (mean ± SD, years)	31.6 ± 11.1	48.2 ± 9.9	<0.001
Donor sex females, Yes (N, %)	29 (60.4)	31 (93.9)	0.001
Recipient sex males, Yes (N, %)	42 (87.5)	32 (97)	0.138
Donor-recipient relation (N, %)	Parents: 34 (70.8)	Wife to husband: 31 (93.9)	
	Siblings: 12 (25)	Husband to wife: 2 (6.1)	
	Children: 1 (2.1)		
	Grandparents: 1 (2.1)		
Socioeconomic status, rural (N, %)	21 (43.8)	14 (42.4)	0.906
Pre-transplant donor eGFR (mean ± SD, mL/minute/1.73m²)	98.8 ± 9.4	97.0 ± 8.8	0.385
Pre-emptive transplant (N, %)	1 (2.1)	3 (9.1)	0.182
Dialysis vintage (mean ± SD, months)	4 (3-7.7)	6 (2-9.5)	0.372
Induction therapy, Yes (N, %)	15 (31.3)	6 (18.2)	0.187
Maintenance immunosuppression (N, %)			0.402
Prednisolone/Tacrolimus/Mycophenolate	39 (81.3)	29 (87.9)	
Prednisolone/Tacrolimus/Azathioprine	4 (8.3)	3 (9.1)	
Prednisolone/Cyclosporine/Azathioprine	4 (8.3)	0	
Prednisolone/Cyclosporine/Mycophenolate	1 (2.1)	1 (3)	
Duration of follow up (mean ± SD, months)	12.2 ± 3.9	12.1 ± 4.1	0.905
Slow/delayed graft function (N, %)	9 (18.8)	7 (21.2)	0.784
BPAR (N, %)	9 (18.8)	6 (18.2)	0.948
Graft dysfunction at six months (N, %)	13 (27)	1 (3)	0.007
Graft dysfunction at one year (N, %)	8 (30.8)	1 (5.9)	0.053
One-year death censored graft survival (%)	85.4	100	0.021
One-year patient survival (%)	93.8	87.9	0.297
Post-transplant infections (N, %)	19 (39.6)	16 (48.5)	0.427
PTDM	14 (29.2)	8 (24.2)	0.624
Post-transplant erythrocytosis (N, %)	6 (12.5)	5 (15.2)	0.489

## Discussion

The salient findings of our prospective observational study of short-term outcomes of live related (60%) and spousal (40%) renal allograft recipients were one-year graft survival, death-censored graft survival, and patient survival rates of 85.2%, 92.6%, and 91.3%, respectively. This is comparable to most other single-center studies as well as large registry data [1-5,7-9] (Table 5).

**Table 5:** Comparison of major studies analyzing the predictors of graft and patient survival in living donor renal transplantation with the current study. *Combined live spousal and live unrelated donors. ATG: anti-thymocyte globulin; AZA: azathioprine; CMV: cytomegalovirus; CSA: cyclosporine; CMV: cytomegalovirus; DM: diabetes mellitus; eGFR: estimated glomerular filtration rate; HLA: human leukocyte antigen; IS: immunosuppressant; MMF: mycophenolate mofetil; PTDM: post-transplant diabetes mellitus; SD: standard deviation; TAC: tacrolimus

First author	Ghoneim et al. [1]	Hassanzadeh et al. [2]	Fuggle et al. [3]	Shahbazi et al. [4]	Mukhopadhyay et al. [5]	Current study
Year of study	1976–2008	1999–2009	2000–2007	2001–2011	2002–2007	2015–2017
Country	Egypt	Iran	UK Tx registry	Iran	Chandigarh, India	
Type of the study	Retrospective	Retrospective	Retrospective	Retrospective	Retrospective	Prospective
Number of patients	1,967	843	3,142	225	554	81
Recipient age (years, mean ± SD, median (range))		35.2 ± 13.4	36 (24–46)	36.4 ± 14.3	33.6 ± 10.3	38.4 ± 13.4
Recipient M: F	2.9: 1	2.2: 1	1.5: 1	1.5: 1	6: 1	10.6: 1
Donor age (mean ± SD, median (range))		32.7 ± 8.6	47 (38-55)	28.8 ± 5.2	42.4 ± 11.3	48.3 ± 9.0
Donor M: F	0.92: 1	1.6: 1	0.82: 1	5.3: 1	0.49: 1	0.35: 1
Type of donor (%)						
Live related	82.3	37.9	71	4.9	76.3	59.3
Live spousal	0	20.5	29*	79.1*	17.2	40.7
Live unrelated	17.7	52.2	29*	79.1*	6.5	0
Induction therapy (%)						
No induction	43.7	-	-	-	90.8	74.1
Basiliximab	0	-	-	-	8.1	25.9
ATG	56.3	-	-	-	0	0
Daclizumab	0	-	-	-	1.1	0
Rejections (%)	48.6	-	-	-	25.6	14.8
One-year death censored graft survival	-	98.3	95	99.1	92	92.6
One-year patient survival	-	-	99	-	94	91.3
Predictors of graft loss						
Advanced donor age	Yes	Yes	Yes	-	-	-
Advanced recipient age	-	-	-	-	Yes	-
Adolescent recipients	-	-	Yes	-	-	-
Female donors	-	Yes	-	-	-	-
Female recipients	-	-	Yes	-	-	-
Low pre-transplant donor eGFR	-	-	-	-	-	Yes
Delayed graft function	-	-	-	Yes	-	-
IS other than TAC-based triple therapy	Yes	-	-	-	Yes	-
Rejections	-	-	-	-	Yes	-
BK virus Nephropathy	-	-	-	-	Yes	-
Creatinine at discharge >2 mg/dL	-	Yes	-	-	-	-
Total steroid dose in three months >5 g	Yes	-	-	-	-	-
Predictors of patient survival						
Advanced donor age	-	-	Yes	-	-	-
Advanced recipient age	-	-	-	-	Yes	-
Unrelated transplants	-	-	-	-	Yes	-
Grafts from offspring to parents	-	-	Yes	-	-	-
PTDM	-	-	-	-	Yes	Yes
Pre-transplant DM	-	-	Yes	-	-	-
HLA-DR mismatch	-	-	Yes	-	-	-
Rejections	Yes	-	-	-	-	-
CMV infection	-	-	-	-	Yes	-

The majority of recipients in our study were males, whereas donors were primarily females. This gender discrepancy is also seen in other studies from the Indian subcontinent and reflects the male-dominated sociocultural environment and is not likely a result of willful gender inequality. However, sex matching did not affect graft or patient outcomes in our study, and most previous studies have shown poor graft outcomes in female donor and male recipient pairs. This may be due to nephron underdosing, increased immunogenicity of female donor kidneys, and increased susceptibility of female allografts to calcineurin inhibitor (CNI) nephrotoxicity [10].

In our study, donor and recipient age were not significant predictors of graft and patient outcomes on multivariate analysis. However, advanced donor age is associated with poor graft survival in large retrospective series [1-3]. The postulated reasons are due to nephron underdosing, increased vulnerability to CNI nephrotoxicity, and accelerated senescence. Advanced donor age is also associated with increased rejection rates and increased mortality [3]. Advanced recipient age is associated with poor graft survival due to age-related factors in the recipient’s serum (lipoprotein and transforming growth factor-β (TGF-β)) which may lead to accelerated senescence of allograft. Patient survival rates are lower in older recipients compared to younger recipients, but higher than in dialysis patients on the transplant waiting list. Age matching has shown better results when older donor kidneys are given to older recipients compared to younger recipients [8].

The most significant predictor of graft loss on multivariate analysis was low pre-transplant donor eGFR (p = 0.046). This was similar to the findings of Norden et al. who reported that death-censored graft survival was significantly lower in grafts from donors with GFR less than 80 mL/minute with a relative risk of graft loss of 2.28 [11]. In a retrospective study involving 206 living donor renal transplants, Hawley et al. reported that pre-transplant donor eGFR was the most significant predictor of six-month recipient graft function [12]. Similarly, in a French study involving 90 donor-recipient pairs, donor age and eGFR were the strongest predictors of recipient kidney function at three years on multivariate analysis [13].

In our study, PTDM was the most significant predictor of mortality on multivariate analysis (p = 0.020). PTDM is a risk factor for both cardiovascular diseases (CVD) and infections, which are the two most common causes of mortality in transplant settings. Using data from the USRDS database, Kasiske et al. also demonstrated that PTDM was associated with an increased risk of graft loss (hazard ratio (HR) = 1.46) and mortality (HR = 1.87) [14]. Pre-transplant chronic HCV infection (p = 0.023) and cyclosporine use (p = 0.024) were the most significant predictors of PTDM. In a retrospective cohort of 557 renal transplant recipients from China, HCV infection was associated with a 3.03-fold risk of PTDM on multivariate analysis [15]. In a meta-analysis involving 2,502 renal transplant recipients from 10 studies, a strong association was found between pre-transplant anti-HCV antibody positivity and PTDM with an adjusted HR of 3.97 [16]. HCV infection is associated with an increased risk of diabetes in the non-transplant population [17]. However, the diabetogenic pathomechanism of chronic HCV infection is not fully understood and involves both increased insulin resistance and reduced insulin secretion. Insulin resistance occurs due to degradation and downregulation of insulin receptor substrate (IRS), alterations in insulin signaling pathways, induction of viral hepatic steatosis, and increase in reactive oxygen species and inflammatory cytokines causing peripheral and hepatic insulin resistance. HCV infection induces the destruction of β cells of the pancreas either directly or through cytokine release leading to reduced insulin secretion [18]. Although PTDM is associated with both tacrolimus and cyclosporine use [19-21], a higher incidence with tacrolimus was observed in the DIRECT randomized controlled trial [22]. Glucose enters adipocytes and striated muscle cells via the GLUT-4 transporter. CNIs downregulate GLUT-4 expression on these cells leading to reduced glucose uptake and hyperglycemia. In addition, they interfere with the signaling of activated T cells in pancreatic β cells leading to a decrease in β cell density and reduction in insulin synthesis [23].

The two most significant predictors of graft dysfunction at six months were rural patients and recipients of related transplants. Almost half of our patients belonged to a rural population with agriculture the main livelihood. We observed that patients of rural populations had a higher incidence of BPARs (p = 0.057) and graft dysfunction at six months (p = 0.040). Non-compliance to medications might be the reason for the adverse graft outcomes. Mittal et al. reported higher rates of BPARs and graft dysfunction among related donors compared to spousal donors in a study involving 323 living donor transplants [24]. The authors postulated lesser use of induction therapy among related transplants as the possible cause. However, we did not observe any difference in the use of induction therapy as well as rejection rates between related and spousal transplants. On the contrary, Fuller et al. observed that rejections were more common in unrelated transplants compared to related transplants which were influenced by a greater number of human leukocyte antigen (HLA) mismatches in unrelated transplants [25]. However, a meta-analysis by Simforoosh et al. concluded that there was no difference in the 10-year graft survival and rejection rates between living related and unrelated transplants [26]. The favorable renal prognosis among spousal renal transplants in our study may be due to lower donor age [44.7 ± 8.0 (spousal donors) versus 50.8 ± 8.9 (related donors); p = 0.02].

Non-use of induction therapy is a cost-saving strategy used by many centers in developing countries [27]. In a randomized controlled trial (RCT) comparing no induction versus induction with basiliximab in 100 live donor renal allograft recipients, no difference was noted in the 10-year graft survival rates. However, BPARs and cumulative steroid dosage were significantly lower in the basiliximab group [28]. We observed that non-use of induction therapy was associated with an increased risk of DGF (p = 0.008), graft dysfunction at six months (p = 0.046), and post-transplant infections (p = 0.037). The higher incidence of post-transplant infections in recipients without induction therapy reflects the higher cumulative steroid dosage and need for ART. ART is associated with a three times higher risk of developing post-transplant infections requiring hospitalization [29]. This undermines the importance of induction therapy even in low-risk transplant recipients.

DGF is reported in 5-10% of living donor kidney transplants (4.9% in our study) [30]. DGF was the most significant predictor of BPAR (p = 0.001) and was associated with an increased risk of graft dysfunction at six months (p = 0.008), post-transplant infections (p = 0.001), and PTDM (p = 0.027). DGF is a consequence of ischemic reperfusion injury caused by a pro-inflammatory cascade that activates toll-like receptors (TLRs) and stimulates the expression of HLA on the graft endothelium fostering an immunological milieu paving the way for rejections [31,32].

UTIs are frequent after kidney transplantation but the impact on short-term outcomes is not well established. In a study by Bodro et al. in 867 kidney transplant recipients, the incidence of UTI and acute graft pyelonephritis was 21% and 15%, respectively. Although uncomplicated UTI was not associated with graft impairment, the development of at least one episode of acute graft pyelonephritis was associated with graft loss at one year [33]. One-third of our cohort developed at least one episode of UTI, with one patient losing his graft due to pyelonephritis.

The prospective longitudinal follow-up over one year after living donor kidney transplantation and a holistic approach to include predictors of all clinically relevant outcomes are the strengths of our study. However, our study is not without limitations. HLA typing and donor-specific antibodies (DSAs) were done pre-transplant in only a minority of patients in view of financial constraints. However, in the era of potent immunosuppressants, the role of HLA mismatches on graft outcomes is controversial [34]. With a paucity of donor organs and the growing epidemic of end-stage kidney disease, rejecting a donor based on the number of HLA mismatches is not justified. Pre-transplant as well as de novo DSAs are important predictors of graft survival [35]. However, in resource-limited settings, we restrict its use prior to high-risk transplants and in the diagnosis and treatment of ABMRs. Moreover, due to logistic reasons, peri-operative predictors such as cold ischemia time and time to diuresis were measured in only a subset of patients and were not analyzed.

## Conclusions

The results of this prospective observational study of short-term outcomes in living donor transplantation of 81 consecutive cases over a one-year period have shown one-year graft survival, death-censored graft survival, and patient survival of 85.2%, 92.6%, and 91.3%, respectively. Graft and patient survival in living donor kidney transplantation are influenced by a multitude of interdependent factors in the pre-transplant (donor eGFR, type of donor, socioeconomic status, HCV infection in recipient, and type of immunosuppression) and the post-transplant (DGF, rejections, infections, and PTDM) period.
